# Evaluation of the promising neighbourhoods community program to reduce health inequalities in youth: a protocol of a mixed-methods study

**DOI:** 10.1186/s12889-019-6901-3

**Published:** 2019-05-14

**Authors:** Mirte Boelens, Dafna A. Windhorst, Harrie Jonkman, Clemens M. H. Hosman, Hein Raat, Wilma Jansen

**Affiliations:** 1 Department of Public Health, Erasmus University Medical Centre, Rotterdam, the Netherlands; 2 Verwey-Jonker Institute, Utrecht, the Netherlands; 3 Department of Health Promotion, Maastricht University, Maastricht, the Netherlands; 4 Department of Clinical Psychology, Radboud University, Nijmegen, the Netherlands; 5 Hosman Prevention and Innovation Consultancy, Berg en Dal, the Netherlands; 6 Municipality of Rotterdam, Rotterdam, the Netherlands

**Keywords:** Youth, Children, Evaluation, Health, Community-based, Collaboration, Stakeholder participation, Socioeconomic prevention

## Abstract

**Background:**

Reducing socioeconomic health inequalities among youth is a major challenge for governments around the world and reports on successful attempts are scarce. Socioecological and integral approaches with collaborative partnerships and community engagement are recommended but knowledge about the effectiveness and effective and ineffective elements is limited. The Promising Neighbourhoods program employs such an approach aiming to reduce socioeconomic inequalities in health, safety and talent development in youth. We will evaluate the process-implementation, and effectiveness of the Promising Neighbourhoods program.

**Methods/design:**

Core elements of Promising Neighbourhoods are a collaborative community programming approach with stakeholders, data-based priority setting, knowledge-, and theory-based policies and evidence-based interventions. Community stakeholders and key-leaders from the neighbourhoods are engaged in the program. For this evaluation study the program will be implemented in three intervention neighbourhoods. These neighbourhoods will be compared to three control neighbourhoods at baseline in 2018/2019 and at follow-up in 2020/2021 after full implementation of the Promising Neighbourhoods program. Intervention neighbourhoods receive a tailored intervention-package including evidence-based interventions and additional measures by community stakeholders. In control neighbourhoods, no special planning will take place thus interventions are offered as usual. A mixed-methods approach following the stages of the logic model from program is applied for this evaluation. Questionnaires, focus groups, and registration data will be collected among community stakeholders, key-leaders, and youth to evaluate the process-implementation of the program. Indicators of intermediate and ultimate outcomes will be studied among *N* = 818 children and *N* = 818 youngsters using difference-in-difference regression analysis to evaluate the effectiveness of the Promising Neighbourhoods program.

**Discussion:**

Hypotheses are that a collaborative community approach with stakeholders leads to clear priority-setting and better tailored interventions of better quality. We further hypothesise a decline in socioeconomic inequalities in intermediate and ultimate outcomes for health, safety and talent development in the intervention neighbourhoods in comparison to control neighbourhoods. The results add knowledge about effective and ineffective elements of collaborative community programming approaches to reduce health inequalities in youth and thus are relevant for local and national public health authorities.

**Trial registration:**

Netherlands National Trial Register number NL7279. Date of registration: 26-Sept-2018.

## Background

Reducing socioeconomic health inequalities is a major challenge for governments around the world and many investments have been made in developing strategies and programs to reduce inequalities [1–4]. However, no convincing reductions in health inequalities at population level have been reported and even an increase is mentioned [5]. Neighbourhood quality, parenting, family life, and the bio-socio-emotional development of youth are thought to explain at least part of the association between socioeconomic conditions and health inequalities [5, 6]. Epidemiological research demonstrated health inequalities in (mental) health status, youth physical activity, school performance, safety, and health related behaviours [7]. In the long term adverse socioeconomic conditions could result in vulnerabilities affecting the perspectives of youth.

To promote health at the local level, socioecological, integral approaches, collaborative partnerships and community engagement are recommended by Weiss et al., and the World Health Organization [7, 8]. Local governments are pivotal in targeting health inequalities, because of their responsibility for social and health policies and their involvement in organising the delivery of social and health services. Furthermore, local governments are in a strong position to bring local actors together for collaboration [9]. At present, knowledge is still limited on how local governments, including municipalities, can successfully implement such integral collaborative community programs, developed in collaboration with community stakeholders. Also, knowledge about what factors determine program successes and failures is still limited [8–10]. The scoping review by Weiss et al.*,* mentions facilitators for successful implementation of collaborative community programs such as the Rural Mental Health project [8]. The Rural Mental Health Project is a promising program targeting mental health, well-being and employment in two rural communities in Northern Ireland [11–13]. The most frequently mentioned facilitating factors are multidisciplinary collaboration, trust between stakeholders, community engagement and inclusion, local planning and action, adequate resources and the use of a dynamic approach [8]. However, most of the studies included in this scoping review are programs targeted at adults, and not much is known about local community programs targeting youth [8].

Two examples of collaborative community programs that are aimed at promoting the health and well-being of youth are Ensemble Prévenons l’Obésité Des Enfants/Together Let’s Prevent Obesity (EPODE) and Communities that Care (CtC) [14–24]. EPODE uses an integrated community-based approach and includes stakeholders at the national and local level [14]. Variations of EPODE are implemented in several countries. Twelve years after initiation of the first EPODE school- and community based program in France, downward trends in overweight prevalence and obesity prevalence were demonstrated [15]. A downward trend was also demonstrated in Belgium but not in Spain [25, 26]. A process evaluation of 18 EPODE programs among several countries demonstrated that good relations between the local project coordinator, program implementers and stakeholders are seen as an important factor for the effectiveness of EPODE [16]. CtC is an integral community prevention coalition aiming to reduce substance abuse and anti-social behaviour among youth and implemented in several countries including the United States of America (USA), United Kingdom and the Netherlands. CtC was evaluated by the Community Youth Development study in seven matched USA states. The participants in CtC communities were more likely to have abstained from cigarettes, alcohol and drugs compared to control communities [18, 23, 24]. In the United Kingdom and the Netherlands, the results were mixed. For the Netherlands disappointing results are explained by shortcomings in internal validity and study design such as lack of tested and effective preventive interventions, contamination and small sample sizes [17, 19, 27–29]. Process evaluations demonstrated that CtC communities had a greater adoption of a science-based approach and used more effective interventions compared to control communities [17, 20–22]. Even though these collaborative community programs showed mixed results they demonstrated some important facilitators for successful implementation, and more needs to be learned about their effective and less effective elements to be able to design effective and integral policies and implementation strategies on the local level [8, 30].

Rotterdam, the second largest city in the Netherlands, has many deprived inner-city neighbourhoods. Around one in five of the children and youngsters in this city are raised in poverty [31]. Reducing poverty and socioeconomic health inequalities are recognized as a major challenge by the municipality [31]. The Promising Neighbourhoods program was developed with the aim to decrease health inequalities and to increase health, safety and talent development among youth. A collaborative community approach with stakeholders, using data-based priority setting, knowledge-, and theory-based policies and with evidence-based interventions in a package tailored to the needs of each specific neighbourhood are the core of the Promising Neighbourhoods program. A thorough process- implementation and effect evaluation of the Promising Neighbourhoods program is set up to disentangle which factors are important for successful implementation of these types of collaborative community approaches and to determine the effectiveness. The Promising Neighbourhoods program will be implemented in intervention neighbourhoods which will be compared to control neighbourhoods. This paper describes the design and methodology of the evaluation study of Promising Neighbourhoods.

### Research questions

The first research question is: how does the process-implementation of the program evolve and what are effective and less effective steps and elements of the Promising Neighbourhoods program? The second research question is: what is the effectiveness of the Promising Neighbourhoods program on reducing socioeconomic inequalities in intermediate outcomes (determinants: protective and risk factors) and ultimate outcomes (health, safety, and talent development) in youth.

### Study hypothesis

We hypothesise that a collaborative community programming approach with stakeholders leads to clear priority setting and better tailored interventions of better quality. Further, we hypothesise a decline in socioeconomic inequalities in intermediate and ultimate outcomes for health, safety and talent development in intervention neighbourhoods in comparison to control neighbourhoods. Intermediate outcomes include the following indicators of risk and protective factors targeted by the program Promising Neighbourhoods: family environment, healthy exercise, nutrition behaviours, smoking and substance use, social cohesion, use of facilities and care and bullying. Ultimate outcomes are indicators of health, safety and talent development.

## Methods/design

### Description of the promising Neighbourhoods program

**Fig. 1:**
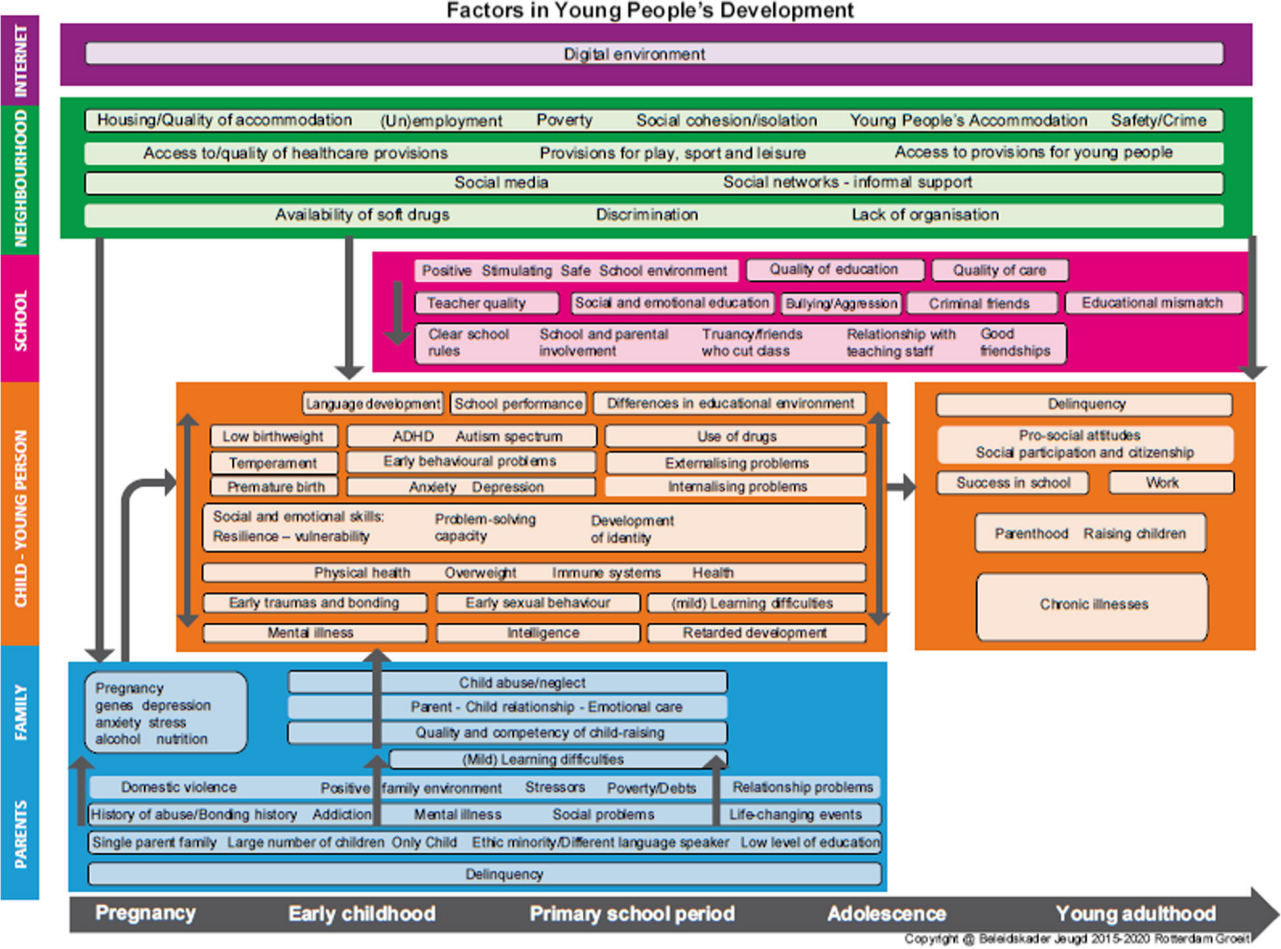
Socioecological framework of the Rotterdam Youth Policy Framework 2015–2020. This socioecological framework provides an overview of parental/family, child-youth, school and community risk and protective factors that influence the development of children from pregnancy to young adulthood onward

The Promising Neighbourhoods program is part of the youth policy ‘Rotterdam Grows 2015-2020’ of the municipality of Rotterdam [32]. This youth policy is based on a multi-level socioecological framework of interrelated risk and protective factors in the life course from pregnancy to young adulthood, including socioeconomic factors (see Fig. 1). This framework provides the theoretical foundation for an integral youth policy program to achieve improvements in several interrelated youth policy domains. The Promising Neighbourhoods program uses this framework and partly builds on experience of methodologies like the community-based EPODE program and the CtC approach [20, 33]. Aims of the Promising Neighbourhoods program are to decrease health inequalities and to increase health, safety, and talent development in youth. The Promising Neighbourhoods program focuses on prevention, stimulation of development, capacity building, empowerment of youth and their families, and improvement of neighbourhood quality, using a collaborative community programming approach with stakeholders, data-based priority setting, knowledge-, and theory-based policies and evidence-based interventions.

The collaborative community programming approach is managed by municipal district advisors. Each municipal district advisor is assigned to a different neighbourhood and will coordinate and monitor the collaborative community programming approach. Every municipal district advisor plans a neighbourhood tailored intervention-package consisting of parenting support, preventive (health) interventions, youth welfare, measures, facilities and activities to improve preventive factors and reduce risk factors for health, safety and talent development among youth. Each neighbourhood receives a tailored intervention-package according to the individual needs of that particular neighbourhood. These needs are assessed using quantitative indicators of the underlying protective and risk factors (corresponding to the theoretical framework) and in consultation with the community stakeholders and key-leaders within the neighbourhood network. The collaborative community programming approach consists of eight steps.

In step one, the needs-assessment of the neighbourhood will be performed using routinely collected data by the municipality of Rotterdam in the so-called Youth Monitor database [34]. This monitor comprises of around 250 quantitative indicators in the areas of health, safety and talent development. The data are collected from various sources, including Statistics Netherlands (CBS), police databases, survey data and routinely collected registration data by health professionals of the Child & Family Centres. These Child & family centres provide basic preventive health services and function as intermediaries for specialized youth care providers. The indicators in the Youth Monitor database correspond to the risk and protective factors in the above-mentioned theoretical framework. The theoretical framework is used by the municipal district advisors to interpret the data and relations between the findings about priorities in the neighbourhood. Based on the outcomes of the needs-assessment, the municipal district advisors suggest what the priorities should be. At the end of this step, the municipal district advisors prepare a first draft of the needs-assessment report. In step two, the draft needs-assessment report of each neighbourhood is discussed with community stakeholders to match the conclusions based on the quantitative data with their daily experiences and to gain local support by setting joint goals. Subsequently, in step three, the needs-assessment report is adapted based on input of the community stakeholders resulting in a final needs-assessment report of the neighbourhood. In step four, the municipal district advisors inventory the currently available measures, interventions, facilities and activities in the neighbourhood and assess their presence in the so-called database Effective Youth Interventions (EYI) of the Netherlands Youth Institute (NYI) [35]. This is a comprehensive database of all nationally available evidence-based interventions for children and youngsters. The municipal district advisors will do this in collaboration with community stakeholders. In step five, outcomes of step four are discussed by the municipal district advisors with community stakeholders to assess which providers and which measures, interventions, facilities and activities are needed to complete the neighbourhood tailored intervention-package. Priority will be given to EYI database of the Netherlands Youth Institute (NYI) [35]. In step six, a detailed neighbourhood intervention-package plan is drawn and all measures, interventions, facilities and activities that will be implemented are described. In step seven, the intervention-package plan including the proposed measures, interventions, facilities and activities is implemented in the neighbourhood. At last, step eight consists of continuous monitoring and revision of the intervention-package performed by municipal district advisors and community stakeholders in the neighbourhood. The monitoring consists of qualitatively and quantitatively evaluating whether intervention-packages in the neighbourhood have the intended results or not, why the results were achieved or not, and what can be done to achieve the previously set goals.

### Intervention neighbourhoods

The Promising Neighbourhoods program will be implemented in three intervention neighbourhoods as described above.

### Control Neighbourhoods

In three comparable control neighbourhoods the Promising Neighbourhoods program will not be implemented during this evaluation study. Preventive measures, interventions, activities and facilities will take place in the control neighbourhoods as usual but there will be no collaborative community programming with stakeholders no data-based priority setting and no promotion of knowledge-, and theory-based policies and evidence-based interventions.

### Evaluation strategy using a logic model

**Fig. 2:**
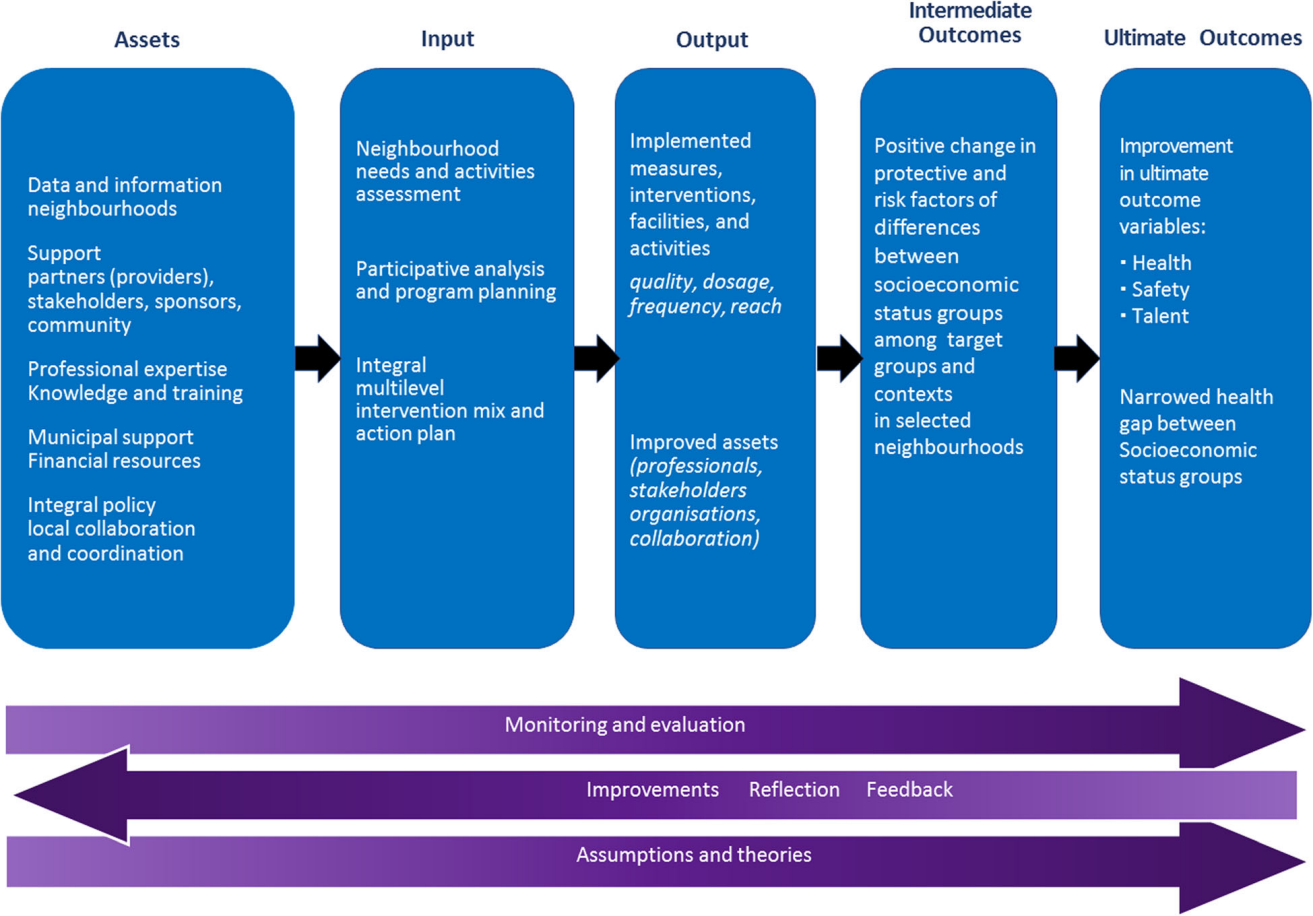
Logic model for the Promising Neighbourhoods evaluation study

To evaluate the Promising Neighbourhoods program, a logic model is used (see Fig. 2). This logic model is used as overall guiding framework for the evaluation study [36]. The logic model contains five stages: assets, input, output, intermediate outcomes and ultimate outcomes. All elements of the logic model, i.e. all different stages of the Promising Neighbourhoods program, will be studied and evaluated. The assets, input and output correspond with the process-implementation evaluation and the intermediate and ultimate outcomes correspond with the effect evaluation. The ‘assets stage’ consists of existing data and information about the participating neighbourhoods, the support from partners, community stakeholders, sponsors, and target groups in the neighbourhoods, municipal support, financial resources, expertise, knowledge, local collaboration and coordination. The ‘input stage’ includes the neighbourhood-specific needs-assessment of the municipal district advisors together with community stakeholders, the assessment of measures, interventions, facilities and activities, already present the in neighbourhood and the intervention-package and action plan. The ‘output stage’ includes the type, number, frequency, intensity, quality, costs and reach of the measures, interventions, facilities and activities and improved assets such as capacity building and empowerment. Targeted intermediate outcomes are improvements in risk and protective factors of youth health, safety and talent development. The targeted ultimate outcomes are improvements of socioeconomic inequalities in health, safety, and talent development among youth in Rotterdam.

### Study design

The study consists of a process-implementation and effect evaluation of the Promising Neighbourhoods program using a mixed-methods design. The process-implementation evaluation corresponds to the assets, input and output of the logic model. Important elements such as reach, dose delivered, dose received and program fidelity from process-implementation evaluation strategies are incorporated in this evaluation [37, 38]. For the process-implementation evaluation, qualitative and quantitative measurements will be performed at baseline (T0) and at follow-up after implementation (T1) of the Promising Neighbourhoods program. Qualitative measurements will be performed using questionnaires and focus groups. Quantitative measurements will be performed using register-data and questionnaires. The effect evaluation corresponds with the intermediate and ultimate outcomes of the logic model. The effect evaluation is based on two separate cross-sectional samples and routinely collected data at baseline and at follow-up after implementation of the full Promising Neighbourhoods program.

### Setting

This study is carried out in six different neighbourhoods in Rotterdam. Neighbourhoods in Rotterdam are categorized as low, middle or high based on the degree of experienced problems. The categorization is based on the percentage of children aged 4 and 12 years old with a high score on the Strengths and Difficulties Questionnaire (SDQ) and the percentage of overweight children in grade two of primary school in the neighbourhoods [34]. The SDQ is a validated questionnaire to measure emotional problems, conduct problems, hyperactivity/inattention, peer problems, prosocial behaviour and total difficulties score [39]. From each category an intervention neighbourhood was selected resulting in three intervention neighbourhoods. Intervention neighbourhoods were matched to control neighbourhoods as much as possible on these two experienced problems and on average socioeconomic status of the neighbourhoods.

### Study population

For the process-implementation evaluation of the Promising Neighbourhoods program, three different groups are distinguished for data collection at baseline as well as at follow-up after the implementation. The first group consists of all community stakeholders in the field of youth (such as teachers, police officers, youth -, health, social -, community -, and sports workers) in each intervention neighbourhood and each control neighbourhood. We want to include all community stakeholders per neighbourhood, and we expect that the size of the population will range between 30 and 50. The second group consists of youngsters (aged ≥12–18 years) in the intervention neighbourhoods, and a comparable group of youngsters in the control neighbourhoods. We will include eight to ten youngsters from intervention neighbourhoods and eight to ten youngsters from control neighbourhoods. The third group consists of key-leaders who are participating in the Promising Neighbourhoods program in each intervention neighbourhood and a comparable group of key-leaders in each control neighbourhood. Key-leaders of the community know the neighbourhood from inside and are for example: police officers, school superintendents, heads of social services agencies, persons knowledgeable about prevention efforts in the community and municipal district advisors managing the process-implementation of the Promising Neighbourhoods program. We will include eight to ten key-leaders per neighbourhood.

For the effect evaluation of the Promising Neighbourhoods Program, the study population consists of *N* = 818 children aged 0 ≤ 11 years old (*N* = 409 at baseline and *N* = 409 at follow-up) and *N* = 818 youngsters aged 12 ≤ 18 years old (*N* = 409 at baseline and *N* = 409 at follow-up), living in the selected intervention and control neighbourhoods in Rotterdam.

### Data collection/outcomes

The data collection that will be performed for this evaluation study is discussed separately for each stage of the logic model. The baseline measurement takes place from November 2018 to February 2019. Implementation of the Promising Neighbourhoods program in the intervention neighbourhoods will take place from February 2019 onwards, after the baseline measurement. The follow-up measurement will take place from September 2020 to March 2021.

### Assets

Data regarding the assets (knowledge and expertise of community stakeholders about the neighbourhood such as existing problems, municipal support, support from partners, sponsors, and target groups, willingness to collaborate in the Promising Neighbourhoods program, and collaboration and coordination in the neighbourhood) will be studied among community stakeholders in all participating neighbourhoods using an anonymous online-questionnaire and focus groups (see Table 1). This online-questionnaire is based on an instrument (Community Board Interview) to collect information about community stakeholders in neighbourhoods and their role in the process-implementation and their characteristics [19]. This instrument was used for evaluation of the CtC approach in the USA, the Netherlands, and Germany [17, 20, 40] and will be adapted for this process-implementation evaluation of Promising Neighbourhoods. The questionnaire will take at most 30 min to fill out and will be administered from November 2018 to February 2019 and from September 2020 to March 2021. Subsequently, focus groups addressing the assets will be organized from November 2018 to February 2019 and from September 2020 to March 2021 in each neighbourhood. In total, there will be six focus groups at baseline and six focus groups at follow-up. Additionally, two focus groups among youngsters (aged 12 ≤ 18 years old) will be organized, both at baseline and at follow-up; one group representing the intervention neighbourhoods and one group representing the control neighbourhoods. The same topics regarding the assets will be addressed. The focus groups among youngsters are specifically used to take the perspective of youngsters about the quality of neighbourhood conditions into account. To assess financial resources, we will use registration data of the municipality of Rotterdam.

**Table 1:** Overview of evaluation measurements

Logic model	Indicators	Measures	Study population/data	Time point	
Process-implementation evaluation				T0	T1
Assets	knowledge and expertise among community stakeholders in the neighbourhood, support from sponsors, willingness to collaborate in a CCP approach, use of interventions^a^, collaboration & coordination in the neighbourhood, financial and municipal resources	online-questionnaire, focus groups, registration data	community stakeholders, youngsters, municipality of Rotterdam	X	X
Input	existing problems in the neighbourhood, effectiveness of community coalitions, decision-making process, existing interventions in the neighbourhood, interventions^a^ proposed by community stakeholders, quality of interventions^a^, quality of the intervention-package of the CCP approach	online-questionnaire, focus groups, registration data	community stakeholders, youngsters, municipality of Rotterdam	X	X
Output	actual implemented interventions^a^, quality of and collaboration of coalitions among community stakeholders, organizations, increase in assets, costs, type, quality, frequency, intensity, reach of interventions^1^, characteristics of the reached groups, use of effective programs, monitoring of implementation and effects	registration data, questionnaire	municipality of Rotterdam, key-leaders	X	X
Effect evaluation					
Intermediate outcomes	indicators of: family environment (parenting, child-parent relationship, family life, family conflict), healthy exercise and nutrition behaviours, smoking and substance use, social cohesion, use of facilities and care, bullying	Health survey (baseline/ similar questionnaire follow-up), You and Your Health (third grade) or SDQ (first grade)	children aged 0 ≤ 11 years old, youngsters aged 12 ≤ 18 years old	X	X
Ultimate outcomes	indicators of: health (socio-emotional and/or psychological problems, general (physical) health, overweight), safety: (home environment, neighbourhood), and talent development: (school performance, truancy)	Health survey (baseline/ similar questionnaire follow-up), You and Your Health (third grade) or SDQ (first grade), routinely collected data	children aged 0 ≤ 11 years old, youngsters aged 12 ≤ 18 years old, municipality of Rotterdam	X	X
Covariates	age, sex, ethnicity, and socioeconomic status indicators: (educational level of the parents when children are at age 0 ≤ 11, educational level of the youngster 12 ≤ 18)	Health survey (baseline/ similar questionnaire follow-up), You and Your Health (third grade) or SDQ (first grade), routinely collected data	children aged 0 ≤ 11 years old, youngsters aged 12 ≤ 18 years old, municipality of Rotterdam	X	X

Overview of indicators, measures, instruments, study population, source of data and measurement moments in the study following the stages of the logic model. Abbreviations: *CCP* Collaborative community program, *SDQ* Strengths and Difficulties Questionnaire.^a^ Also includes measures, facilities and activities

#### Input

Data regarding the input (effectiveness of neighbourhood networks consisting of community stakeholders in the neighbourhood, and existing measures, plans, interventions, facilities and activities) will be studied using the same online-questionnaire and focus groups in 2018/2019 and 2020/2021 as will be used to study the assets (see Table 1). The anonymous online-questionnaire and focus groups will be held among community stakeholders in all participating intervention and control neighbourhoods. Additionally, in the two focus groups among youngsters representing the intervention neighbourhoods and youngsters representing the control neighbourhoods (aged 12 ≤ 18 years old) the same topics regarding the input will be discussed. To assess existing measures, interventions, facilities and activities in the neighbourhood, we will use registration data of the municipality of Rotterdam and additional surveys of key-leaders.

#### Output

Data regarding the output (costs, type, number, quality, frequency, intensity, reach and characteristics of the reached groups of the implemented measures, interventions, facilities, and activities, use of a knowledge/science-based approach, use of effective programs and monitoring of implementation) will be studied using a questionnaire (see Table 1). This questionnaire will be administered among key-leaders in November 2018 to February 2019 and from September 2020 to March 2021. This questionnaire is an adapted version of the Key-Informant questionnaire previously used in CtC evaluations in the USA, the Netherlands and Germany [17, 22]. This questionnaire will take at most 60 min to fill out and will be administered via telephone. During the focus groups with community stakeholders in 2020/2021, and the online-questionnaire prior to it, the contribution of assets and input to the realized output will be discussed. To examine the costs, type, number, quality, frequency, intensity, reach and characteristics of the reached groups of the implemented measures, interventions, facilities, and activities we will use registration data of the municipality of Rotterdam. These data are registered by the municipal district advisors and providers of the interventions (see Table 1).

#### Intermediate outcomes

In this study, the intermediate outcomes are the proximal results of the measures, interventions, facilities and activities that will be implemented in the neighbourhoods. Therefore, we will study indicators of the following targeted risk and protective factors among children and youngsters: family environment (parenting, child-parent relationship, family life, and family conflict), healthy exercise, nutrition behaviours, smoking and substance use, social cohesion, use of facilities and care, and bullying (see Table 1). Data on intermediate outcomes will be collected at two time points, in 2018 at baseline and in 2020/2021 at follow-up, separately for children aged 0 ≤ 11 years old and for youngsters aged 12 ≤ 18 years old.

For the baseline measurement of intermediate outcomes among 0 ≤ 11 years old children, we will use anonymous survey data from the Health survey obtained in 2018 by the municipality of Rotterdam. The Health survey is administered every four years in a random sample drawn from the municipal population register using online parent-questionnaires. The Health survey consists of questionnaires addressing the following topics: general health, nutrition and exercise behaviour, home-environment, emotional and psychological health, neighbourhood-, and school environment, use of care and facilities, smoking and alcohol [39]. Follow-up data will be collected by administering a comparable parent-questionnaire for children aged 0 ≤ 11 years old in 2020/2021. This questionnaire will be based on the Health survey and will include similar questions. We will use the same procedures for sample selection and administration of the follow-up questionnaire, as used by the municipality for the Health survey (the baseline data).

For the baseline and follow-up measurements in the group of 12 ≤ 18 years old youngsters we will use survey-data obtained by the Child and Family Centre Rijnmond in 2018 and 2020/2021 respectively. These survey data consist of the SDQ and the so-called You and Your health questionnaire which includes several validated questionnaires [39, 41–43]. The You and Your health questionnaire addresses the following topics: general health, nutrition and exercise behaviour, home-environment, emotional and psychological health, school environment, performing anxiety and learning behaviour, smoking, alcohol, substance use and gaming. These questionnaires are routinely administered by the Child and Family Centre Rijnmond every year for municipal health monitoring. The questionnaires are filled out online at school, the SDQ in first grade and You and your Health in third grade.

#### Ultimate outcomes

Ultimate outcome measures are: indicators of health (socio-emotional and/or psychological problems, general/physical health, overweight), safety (safety of the home environment, safety of the neighbourhood) and talent development (school performance, truancy) (see Table 1). Ultimate outcomes will be collected at the same two time-points, at baseline and at follow-up after implementation of the Promising Neighbourhoods program. The same instruments as for the intermediate outcomes will be used, separately for children aged 0 ≤ 11 years old and for youngsters aged 12 ≤ 18 years old. Since truancy, school performance and safety in the neighbourhood are not administered by the Health survey, SDQ or You and Your Health questionnaire we will use routinely collected data on the individual, school and neighbourhood level from the municipality of Rotterdam for these outcomes.

#### Covariates

In addition to the intermediate and ultimate outcomes, demographical data including neighbourhood, age, sex, ethnicity, and socioeconomic status will be collected for the effect evaluation. Socioeconomic status will be measured as the highest educational level obtained either by both parents or the mother for children aged 0 ≤ 11 years old and current educational level of youngsters aged 12 ≤ 18 years old. Educational level will be classified according to the International Standard Classification of Education 2011 [44]. Demographical data will be obtained from the municipality of Rotterdam and the Child and Family Centre Rijnmond.

### Power considerations

The size of the data samples needed to determine small-effect sizes (f2 = 0.02) is calculated for the effect evaluation [45]. Analyses will be performed separately for children aged 0 ≤ 11 years old and for youngsters aged 12 ≤ 18 years old as outcome-variables of interest might differ between age groups and are measured in a different way. Based upon 5% two-sided significance tests of the null hypothesis that socioeconomic status-groups in the intervention and control neighbourhoods do not differ on outcome variables at follow-up and a power of 80% allowing for 10 independent variables in the model, we need two samples of 818 individuals, one sample including children aged 0 ≤ 11 years old and one sample including youngsters aged 12 ≤ 18 years old. Half of each sample will consist of individuals at baseline, evenly distributed over the conditions (intervention or control neighbourhood). The other half will consist of individuals at follow-up, evenly distributed over conditions as well. For the baseline measurement of both age groups, we will have sufficient survey data. For the follow-up measurement of youngsters aged 12 ≤ 18 years old sufficient survey data is available because the Child and Family Centre Rijnmond administers their survey every year. However, since the Health survey of the municipality of Rotterdam is administered only once every four years we will not have sufficient follow-up data for children aged of 0 ≤ 11 years old. Therefore, we will gather these data by administering an additional comparable parent-questionnaire. With a predicted response rate of 40% we will need to reach 1025 parents in order to receive 409 follow-up questionnaires.

### Data analyses

The process-implementation evaluation consists of the assets, input and output stages in the logic model. All focus groups and questionnaires will be documented and analysed using standardized formats. The qualitative data will be analysed using a set of codes. At the start of the analysis, a codebook will be made with herein a list of pre-set codes on different levels such as concepts or categories. This codebook will be made by two researchers independently coding the first two focus groups. During the analysis new codes may occur. These codes will be added to the codebook. Questionnaire data and register-data per intervention and per neighbourhood about the costs, type, number, quality, frequency, intensity, reach, and characteristics of the reached groups will also be analysed to detect changes between the baseline measurement and the follow-up measurement. Intervention and control neighbourhood results will be compared. Because of the relative small size of the groups in the process-implementation evaluation, T-tests with Bayesian statistics will be used [46, 47]. This will be performed using the program Jags in R. Intervention and control estimates will be compared, each chain with 5000 iterations and each time 500 not used. At the end, the estimates will be made with a total of 4500 estimates.

In the effect evaluation of the Promising Neighbourhoods program, effects on the dependent intermediate outcomes (indicators of risk and protective factors) and ultimate outcomes (indicators of health, safety and talent development) of the logic model will be examined. We will perform Difference-in-Difference regression analyses separately for children aged 0 ≤ 11 years old and for youngsters aged 12 ≤ 18 years old. Predictor variables of interest are time of measurement (baseline/follow-up), condition (intervention/control), socioeconomic status, and their two-way and three-way interactions controlled for neighbourhood conditions. We will also adjust for other sociodemographic covariates. The main variable of interest in our study is the three-way interaction term for time of measurement, condition, and socioeconomic status. A significant parameter (*p* < 0.05) for this interaction term indicates a change in outcome over time in the intervention neighbourhoods with a socioeconomic status gradient. In case of continuous outcome variables linear regression analysis will be used and for dichotomous outcome variables logic regression analysis will be used. Missing data will be handled using multiple imputation. Level of significance is set at 0.05 for two-tailed analyses.

## Discussion

This article describes the design and methodology of a mixed-methods study for evaluation of the process-implementation and effectiveness of the Promising Neighbourhoods program. Promising Neighbourhoods is a program aiming to reduce socioeconomic health inequalities and to increase health, safety and talent development among youth in different neighbourhoods of Rotterdam. The Promising Neighbourhoods program will be gradually implemented in all neighbourhoods in Rotterdam, if proven effective.

We hypothesise that a collaborative community programming approach with stakeholders leads to clear priority setting and better tailored interventions of better quality. Furthermore, we hypothesise a decline in socioeconomic inequalities in intermediate and ultimate outcomes for health, safety and talent development in intervention neighbourhoods in comparison to control neighbourhoods. As we also study the process-implementation of the Promising Neighbourhoods program, we will be able to provide relevant insights on possible facilitators and barriers for future implementation of policy programs using a collaborative community approach, such as Promising Neighbourhoods.

A strength of this study is that we study the process-implementation as well as the effectiveness [48]. Without evaluation of the process-implementation, the black box of why a program is effective or not effective cannot be disentangled [48]. Furthermore, the process-implementation evaluation can also shed light on possible defects or unwanted side effects within the Promising Neighbourhoods program [10]. Adding to this, it may elucidate how to successfully adapt programs and reach specific communities. Therefore, this study is relevant for local settings and collaborative programming and for national governments that depend on successful local implementation of policies. Another strong aspect is that we evaluate the process-implementation and effectiveness in different neighbourhoods increasing the generalisability of our findings.

The limitations that need to be taken into consideration are first that long term effects are not part of our study design. The time of two years between the baseline and follow-up does not allow studying longer term outcomes on especially the intended ultimate outcomes in the current study. Second, there is always a chance for residual confounding even though we will adjust for confounding in our analyses and tried to match intervention neighbourhoods as much as possible based on the degree of experienced problems and on socioeconomic status. We cannot control planned or unplanned implementation of interventions in the neighbourhoods on the initiative of other institutions or in some cases even of collaborating community stakeholders. Moreover, we cannot control for the fact that children, youngsters and their families in control neighbourhoods may receive or take part in interventions or activities provided in the intervention neighbourhoods. Or that they take part in interventions or activities implemented independently of interventions that are part of the Promising Neighbourhoods program. However, to monitor this, we will obtain registration data about implemented measures, interventions, facilities and activities in the neighbourhoods.

In conclusion, this study will provide knowledge about the process-implementation and effectiveness of a collaborative community programming approach implemented together with stakeholders, using data-based priority setting, knowledge-, and theory-based policies and evidence-based interventions. The results following from this study may be used for the design, implementation and transferability of intervention programs aiming to reduce health inequalities among youth using a collaborative community programming approach with stakeholders. Therefore, our study is relevant for local and national public health authorities and for improvement of the health, safety, and talent development among youth.

## Abbreviations

CCP: Collaborative community program
CtC: Communities that Care
EPODE: Ensemble Prévenons l’Obésité Des Enfants/Together Lets Prevent Obesity
EYI: Effective Youth Interventions
ICJME: International Committee of Medical Journal Editors
NYI: Netherlands Youth Institute
SDQ: Strengths and Difficulties Questionnaire
T0: baseline measurement
T1: Follow-up measurement
USA: United States of America
